# Human papillomavirus infection and p16 expression in the immunocompetent patients with extragenital/extraungual Bowen’s disease

**DOI:** 10.1186/s13000-016-0505-3

**Published:** 2016-06-24

**Authors:** Marián Švajdler, Roman Mezencev, Jana Kašpírková, Denisa Kacerovská, Dmitry V. Kazakov, Ondrej Ondič, Michal Michal

**Affiliations:** Šikl’s Department of Pathology, Charles University in Prague, The Faculty of Medicine and Faculty Hospital in Pilsen, Pilsen, Czech Republic; Bioptická laboratoř, s.r.o., Mikulášske nám. 4, 326 00 Pilsen, Czech Republic; Integrated Cancer Research Center, School of Biology and Parker H. Petit Institute of Bioengineering and Biosciences, Georgia Institute of Technology, Atlanta, GA USA

**Keywords:** Bowen’s disease, Human papillomavirus, p16, Skin cancer

## Abstract

**Background:**

The role of human papillomaviruses (HPV) in the development of squamous cell carcinoma (SCC) has been established for anogenital lesions but still remains controversial for carcinomas in other sites. The aim of this study was to determine the α-HPV and β-HPV prevalence and their association with p16 expression, sun exposure, and clinicopathological findings in patients with Bowen’s disease (BD).

**Methods:**

One hundred sixty nine skin biopsy specimens from 157 immunocompetent patients with extragenital/extraungual BD were examined for HPV status and p16 expression. The presence of koilocyte-like changes, solar elastosis and papillomatosis was recorded for each specimen.

**Results:**

BD was diagnosed more often in potentially sun-exposed sites with prevalence 73.6 % and a remarkable predilection for the head and neck region. High risk α-HPV or β-HPV were detected in 34.7 % of lesions and β-HPV infections dominated over α-HPV. Higher prevalence of koilocyte-like changes and papillomatosis was found in HPV-positive specimens but it was not statistically significant. The expression of p16 was detected in 79.8 % of lesions and displayed no correlation with the HPV status. HPV-positivity tended to be detected more often in sun-protected sites. Dual infections by α-HPV/β-HPV genera and mixed α-HPV infections were not detected, while 37.5 % of β-HPV positive specimens were infected by two or more β-HPV genotypes. HPV 9 was significantly associated with mixed β-HPV infections.

**Conclusions:**

HPV may play an etiological role at least in some SCC in situ arising in extragenital sites. Sunprotected sites may be more dependent on HPV-mediated co-carcinogenesis than sun exposed areas. The presence of the p16-expression, papillomatosis or koilocyte-like change is not a reliable marker of HPV infection in SCC in situ.

**Electronic supplementary material:**

The online version of this article (doi:10.1186/s13000-016-0505-3) contains supplementary material, which is available to authorized users.

## Background

There is increasing evidence supporting the role of human papillomavirus (HPV) in the development of premalignant and malignant skin lesions in both immunocompetent and immunocompromised patients [1]. However, in contrast to anogenital HPV-associated lesions, where the oncogenic role of mucosal α-HPV genotypes is well established, the role of HPV (especially of genus β-HPV) in extragenital malignancies of the skin is not entirely understood [2]. This is, at least in part, caused by a highly variable prevalence of HPV-positivity observed in invasive carcinomas (basal and squamous cell carcinoma) and pre-malignant squamous skin lesions (such as Bowen’s disease or actinic keratosis) depending on the ethnicity of the study population and methods used for the HPV detection [3–17]. In addition, a relatively high prevalence of β-HPV infections in the normal skin has been reported for some populations, although the difference in the presence of specific β-HPV genotypes has been noted between cutaneous squamous cell carcinoma (cSCC) and normal skin specimens [10]. Nevertheless, the role of β-HPV in skin carcinogenesis is supported by a strong association between specific β-HPV genotypes, especially HPV5, HPV8, HPV14, and HPV20 and the development of cSCC in patients with epidermodysplasia verruciformis (EV), a rare autosomal recessive disorder classified as a primary immunodeficiency [18].

Bowen’s disease (BD) is a form of cSCC in situ (SCCIS), which was originally described by John Templeton Bowen in 1912 in sun-protected sites (these lesions were possibly arsenic-induced) [19]. At the present time, BD is synonymous with non-genital squamous cell carcinoma in situ on sun-protected as well as sun-exposed sites, histologically characterized by full-thickness dysplasia of the epidermis, sometimes with involvement of follicular epithelium, similar to that observed in HPV-induced anogenital intraepithelial neoplasia [20]. In HPV-associated carcinomas, viral oncoprotein E7 expressed by high-risk α-HPV genotypes functionally inactivates the retinoblastoma protein (pRb), which in turn leads to the overexpression of the p16^INK4A^ (p16) tumor suppressor [21] and the immunohistochemical detection of p16 is often used as a surrogate marker for the high-risk HPV infection in squamous cell carcinomas of the vulva, penis, perianal area, uterine cervix and oropharynx [21, 22]. Similarly, the over-expression of p16 was also reported in BD [13, 23–30]; however, only few studies have focused on a correlation of the expression of p16 with the HPV status in Bowen’s disease and when this was performed, a correlation between p16 expression with the presence of mucosal HPV types only was studied [13, 23, 24].

In this study we evaluated by the DNA polymerase chain reactions (PCR) and hybridization the presence of mucosal (α-papillomavirus genus) and cutaneous/epidermodysplasia verruciformis (β-papillomavirus genus) HPV genotypes in 169 biopsy specimens from extragenital/extraungual BD lesions diagnosed in 157 Eastern European Caucasian immunocompetent patients. In addition, we examined the association between the presence of α-HPV, β-HPV and the expression of p16 protein, as well as clinicopathological patterns and the distribution of specific HPV genotypes in BD. Our results provide additional insight into the role of sun-exposure, HPV-infection and development of Bowen’s disease.

## Methods

### Patients, tissue specimens and p16 immunohistochemical staining

**Fig. 1:**
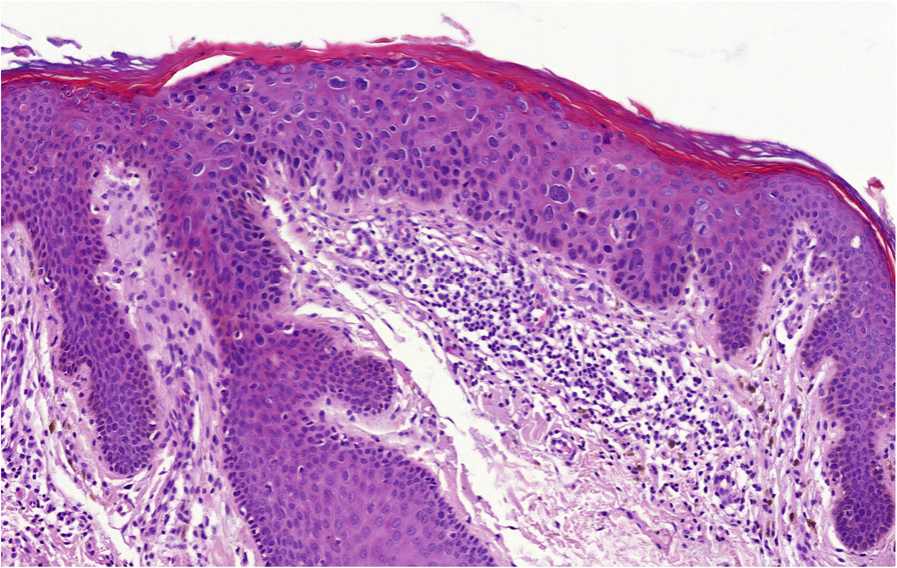
Representative case of Bowen’s disease, with full-thickness involvement of the epidermis by dysplastic keratinocytes. Solar elastosis and chronic inflammation are present in the superficial dermis. Hematoxylin-eosin, original magnification × 200

**Fig. 2:**
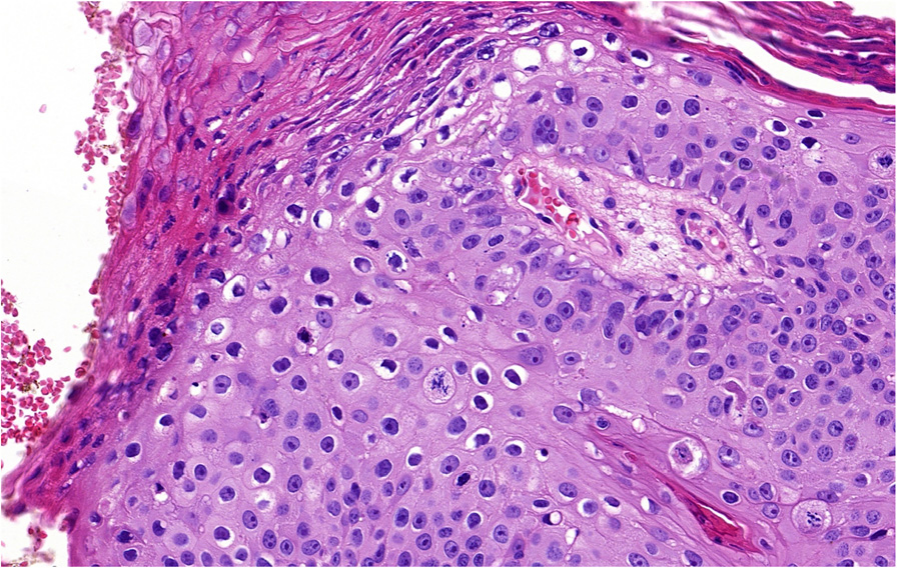
Koilocyte-like change in the superficial part of dysplastic epithelium, characterized by perinulear halo around hyperchromatic and irregular nuclei. Hematoxylin-eosin, original magnification × 400

**Fig. 3:**
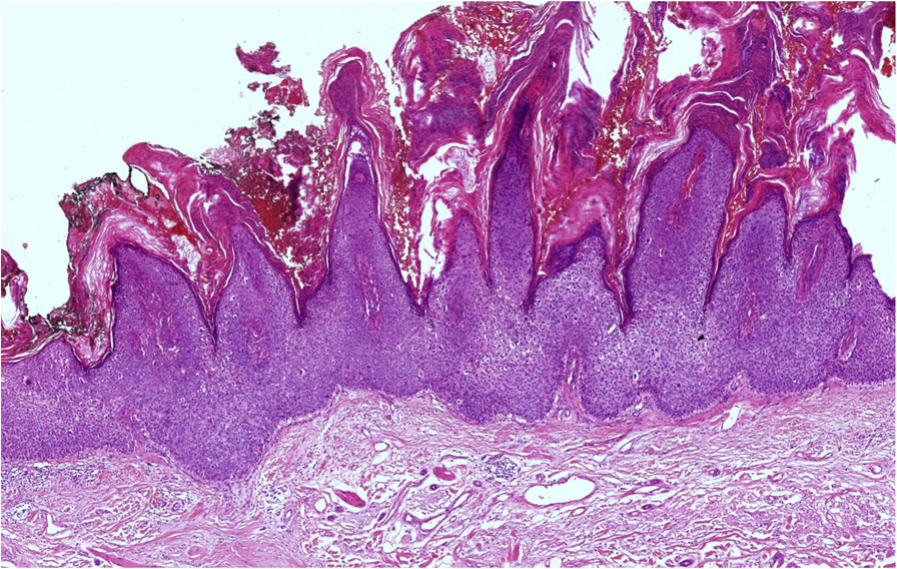
Some Bowen’s disease lesions showed papillomatosis, which was marked in this case. Hematoxylin-eosin, original magnification × 50

**Fig. 4:**
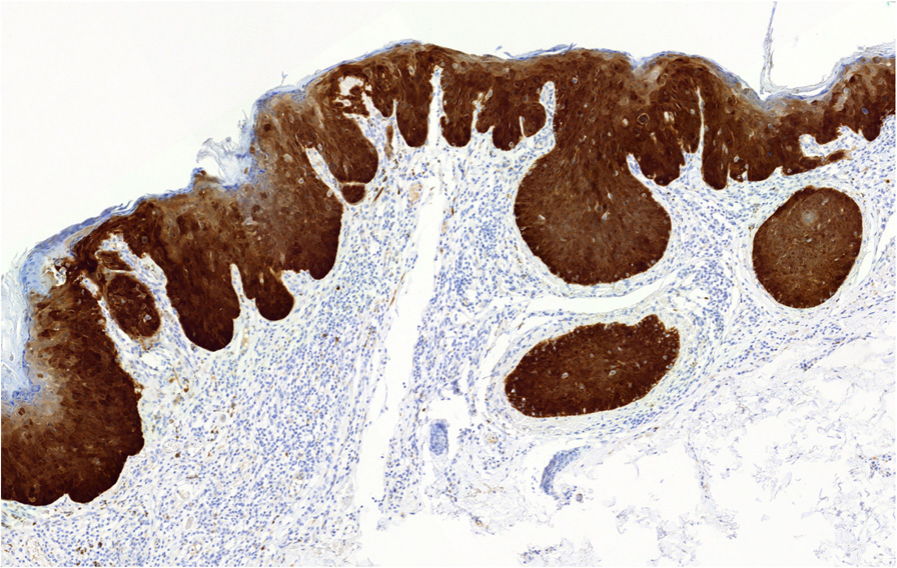
Representative case of p16-positive Bowen’s disease showing diffuse “block-type” nuclear and cytoplasmic p16 staining. Original magnification × 100

After Faculty Hospital in Pilsen Ethical Board approval, a total of 169 skin biopsy specimens collected between 1996 and 2014 from 157 patients diagnosed with Bowen’s disease/SCC in situ were randomly retrieved from the pathology files of Šikl’s Department of Pathology in Pilsen, Czech Republic. None of these patients was diagnosed with an immunosuppressive condition or treated with immunosuppressive drugs. In all these cases, the hematoxylin-eosin stained slides were reviewed to confirm the diagnosis (Fig. 1). The presence of koilocyte-like changes (Fig. 2), solar elastosis and papillomatosis (Fig. 3) was recorded for each specimen. Considering possibility of their different etiological origin, cases of actinic keratosis, Bowenoid type, were not included in the study. These cases usually showed at least focal superficial keratinocyte maturation (the lack of full-thickness atypia), variable (slight to pronounced) atypia of the basal layer keratinocytes at the periphery of the lesion, less defined transition to normal epidermis, and less frequent follicular and acrosyringeal involvement. Likewise, equivocal cases, which were hard to classify between Bowen’s disease/SCC and actinic keratosis, Bowenoid type, were not included. For the detection of p16 by immunohistochemistry (IHC), the most representative paraffin blocks were selected and 4 μm tissue sections were stained with the p16 antibody (CINtec® p16 Histology, Ventana) using the Ventana Benchmark automated stainer, according to the manufacturer’s protocol. Appropriate positive and negative control slides were used. The staining pattern for p16 was classified as positive when showing the “block-type” diffuse nuclear and cytoplasmic staining (Fig. 4), typically seen in HPV-induced anogenital intraepithelial neoplasia. Other staining patterns were classified as p16-negative and included the following findings: (i) negative (no positive cells), (ii) equivocal (weak and focal positivity), and (iii) focal (strong unequivocal positivity in the minority of tumor cells, typically in a map-like pattern).

### Polymerase chain reaction

For molecular-genetic studies, genomic DNA was isolated from formalin-fixed, paraffin-embedded tissue using the QIAsymphony DNA Mini Kit (Qiagen, Hilden, Germany) on QIAsymphony SP device according to the manufacturer’s protocol. Special precautions were taken to prevent contamination of specimens by the HPV DNA. The quality of isolated DNA was examined by the PCR reaction that amplifies set of control genes with different amplicon sizes (100–600 bp) [31].

The detection α-HPV DNA was performed using multiple PCR primers from the L1, E1, and E6–E7 regions of the HPV genome. In brief, primers GP5+/GP6+ targeting L1 region of the HPV genome were used for the standard wide-range detection of mucosal HPVs from α- genus. Type-specific PCR detection of E6-E7 region of the most prevalent six HR-HPV types, namely types 16, 18, 31, 33, 35, and 45, was used to increase the sensitivity of detection for mucosal high risk (HR) α-HPV genotypes and to avoid negative findings due to the possible integration of HPV DNA into the human genome [32].

Cutaneous HPV types were detected by the following two methods: (i) RHA Kit Skin (beta) HPV assay (Diassay B.V., Rijswijk, the Netherlands), which is based on PCR and reverse hybridization of amplified products on a probe-covered strip, was used to detect clinically relevant infection of betapapillomavirus genotypes, namely HPV 5, 8, 9, 12, 14, 15, 17, 19, 20, 21, 22, 23, 24, 25, 36, 37, 38, 47, 49, 75, 76, 80, 92, 93 and 96 and (ii) PCR assay using the CPSGB primers that amplify part of the E1 region of most mucosal α-HPVs as well as cutaneous β- and γ-HPVs [33].

All PCR reactions were run in GeneAmp PCR System 9700 (PE/Applied Biosystem, Forster City, CA). Amplicons were analyzed by electrophoresis in 2 % agarose gel using ethidium bromide staining. Positive PCR samples were genotyped by hybridization to the type specific probes (RHA Kit), or sequenced and compared to the relevant sequences in the NCBI sequence database (GP5+/GP6+ and CPSGB based PCR). Positive and negative controls were included in every single run.

### Data processing and statistical analysis

Based on their anatomical location, lesions were classified as localized in sun-protected (trunk, lower extremities) or potentially sun-exposed (all other sites), excluding anogenital and periungual lesions. For the assessment of anatomical distribution of BD lesions, body surface area corresponding to these sites was considered as follows: head and neck: 9 %; upper extremities: 18 %; trunk: 37 % and lower extremities: 36 % [34].

BD cases that displayed strong diffuse IHC staining pattern for p16 were coded as p16-positive and all other staining patterns were coded as p16-negative. From a molecular standpoint, cases were labeled as HPV-positive if they displayed positivity for the high risk α-HPV or at least one of the two tests used for the detection of β-HPV DNA proved positive. Cases were scored as HPV negative if all 3 assays for high risk α-HPV as well as β-HPV genotypes revealed no virus while displaying no problems in the quality or quantity of the isolated DNA.

Upper and lower limits of 95 % confidence intervals for proportions (CI_95_) were calculated by Wilson method with continuity correction [35]. Significance of differences between sample proportions and hypothesized population proportions was tested using one-sample z-test. Significance of association between two categorical classifications was tested by Fisher’s exact test. The strength of associations between two binary variables (HPV DNA status and p16 expression) was determined by phi coefficients using the package ‘psych’ [36]. in R software [37]. Agreement between the two assays used for the detection of β-HPV DNA was determined by Cohen’s kappa coefficient using the GraphPad QuickCalcs calculator (http://www.graphpad.com/quickcalcs/). All *p*-values are two-tailed unless specified otherwise and the differences were considered as statistically significant if *p* < 0.05).

## Results

The study group consisted of 157 patients of Caucasian race and Eastern European origin (70 females and 87 males) previously diagnosed with Bowen’s disease/SCC in situ. Among them, nine patients developed multiple BD lesions, which were all included in this study (7 patients developed 2 lesions, 1 patient 3 lesions, and the remaining patient 4 lesions). The age at diagnosis (or diagnosis of the first lesion for cases with multiple neoplasms) ranged from 43 to 96 years (median: 74 years for both genders; 76 years for women and 73 years for men). The age at the first diagnosis was not significantly different between the male and female patients (*p* = 0.5972).

From these 157 patients, one or more biopsy specimens corresponding to distinct BD lesions were collected, with a total of 169 biopsy specimens (Additional file 1). The distribution of BD sites was as follows: head and neck region: 58.6 %, lower extremities: 14.8 %; upper extremities: 10.7 %; trunk (chest and back): 10 %; unknown location (but not anogenital area): 5.9 %. The distribution of BD lesions with a known location was not significantly different between male and female specimens. Likewise, no significant differences were identified for other demographic or clinical factors between male and female biopsy specimens. (Additional file 1).

BD was diagnosed more often in potentially sun-exposed sites (in 117 out of 159 lesions with a known location) with prevalence 73.6 % (CI_95_: 66.2–79.8 %), which is significantly higher than 27 % (*p* < 0.0001; one-sample z-test) that would be expected under the assumption of equal disease distribution between sun-exposed and sun-protected sites (corrected for the corresponding body surface areas; see Methods section) and equal average size of lesions in sun-exposed and sun-protected sites. The majority of the lesions from sun-exposed sites are located in the head and neck region (99/117, 84.6 %), while the head and neck region represents only about 1/3 of the total surface of the sun-exposed body area. This indicates a remarkable predilection of Bowen’s disease for the head and neck region over other sun-exposed sites (upper extremities) (*p* < 0.0001; one-sample z-test).

Solar elastosis was found in 93 of 169 biopsy specimens (55.0 %; CI_95_ = 47.5–62.3 %). Among 159 lesions with a known location, solar elastosis was found in 80/117 (68.4 %; CI_95_ = 59.7–76.1 %) lesions from sun-exposed sites but only in 11/42 (26.2 %; CI_95_ = 15.3–41.1 %) lesions from sun-protected sites. The difference between these two proportions is statistically significant (*p* = 0.0001; Fisher’s exact test), which supports the hypothesis that prevalence of solar elastosis is higher in sun-exposed sites. This observation, while somewhat predictable, indicates that our classification of specimens according to the sun-exposure status are correct. In both sun-exposed and sun-protected sites the proportion of BD specimens positive for solar elastosis is notably lower than 100 %, which suggests that the development of BD may require lower cumulative sun exposure than solar elastosis.

Among 168 lesions immunohistochemically evaluable for p16 expression, 134 lesions (79.8 %; CI_95_ = 73.1–85.1 %) were scored as p16-positive. Of the remaining 34 lesions scored as p16-negative, 26 lesions displayed either negative or equivocal/weakly positive staining by IHC and 8 lesions showed p16-positivity that was unequivocal but limited to only a minority of neoplastic cells (map-like staining pattern). As a result, p16-positive cases likely represent the majority of BD cases in general population, with the true proportion of p16-positivity in BD cases significantly higher than 50 % (one-tailed *p* < 0.0001; one-sample z-test).

DNA from 46 biopsy specimens displayed low quality, low quantity, or both. Among them, 42 specimens displayed negative results for HPV DNA by all of the three HPV assays used in this study. Due to the low confidence in these negative results, which may in some cases reflect degradation of analytical target rather than the true absence of HPV DNA, these 42 specimens were not considered in further analyses.

Thus, 127 specimens were evaluable for the presence of α-HPV genotypes, and 13 (10.2 %; CI_95_ = 6.1–16.7 %) were found to be α-HPV positive, including 12 specimens (9.4 %; CI_95_ = 5.5–15.8 %) positive for high-risk α-HPV genotypes (HPV 16: 6 cases; HPV 33: 2 cases; HPV 18, HPV 56, HPV 58, and HPV 66: 1 case each) and one case positive for a low-risk α-HPV genotype (HPV 81). All α-HPV-positive cases (100 %, CI_95_ = 75.8–100 %) and 93/114 α-HPV-negative cases (81.6 %; CI_95_ = 73.5–87.6 %) were found positive for p16 expression by IHC.

Of the seven female patients with BD lesions positive for high-risk α-HPV, cervical cytology and/or biopsy specimens from the uterine cervix were available in four patients. Among them, three patients were negative for intraepithelial cervical lesions or abnormal cytology and one was positive for low-grade squamous intraepithelial lesion (CIN I/LSIL) with high-risk HPV detected by PCR (HPV genotype was not determined). Consequently, HR α-HPV-infected BD lesions appear not to be necessarily accompanied with α-HPV-induced cervical lesions.

Positivity for β-HPV genotypes was detected by two assays, of which RHA Kit Skin (beta) HPV assay, which combines PCR with DNA hybridization, is expected to be more specific for β-HPV at clinically relevant viral loads than E1-based PCR using CPSGB primers (see Methods section). After exclusion of (i) the 42 HPV-negative specimens with problematic DNA (see above) and (ii) 6 cases negative for α-HPV that were found not evaluable for β-HPV, a total of 121 specimens were evaluated for the associations between the combined HPV status (α and/or β genera) and other variables, such as sex, sun exposure, p16 status, and the presence of solar elastosis or koilocyte-like changes (Table 1).

**Table 1:** Crosstabulation of p16-expression and other findings based on the HPV DNA status in 121 biopsy specimens evaluable for HPV DNA

	DNA positive for HR α-HPV or β-HPV	DNA negative for HR α-HPV and β-HPV	*p*-value (Statistical test)
IHC p16			0.4247 (Fisher’s exact test)
positive	34	68	
negative	8	10	
not evaluable	0	1	
Sex			0.7039 (Fisher’s exact test)
F	21	43	
M	21	36	
Site			0.3560 (Fisher’s exact test)
Sun-exposed	28	59	
Sun-protected	11	15	
Unknown	3	5	
Solar elastosis			0.3349 (Fisher’s exact test)
positive	21	48	
negative	21	31	
Koilocyte-like changes			0.1781 (Fisher’s exact test)
positive	21	29	
negative	21	50	
Papillomatosis			0.3170 (Fisher’s exact test)
Positive	17	25	
Negative	24	54	
Unknown	1	0	

The number of specimens infected by β-HPV genotypes was found to be 30/120 (25.0 %, CI_95_ = 18.1–33.4 %) by the E1-based PCR assay and 19/119 (16.0 %, CI_95_ = 10.5–23.6 %) by the RHA Kit Skin (beta) assay. We have also evaluated the concordance of the results of the two β-HPV assays on the 119 cases that were evaluable with both these assays (one case positive by E1-based PCR assay and negative by RHA Kit Skin (beta) was not included due to the low quality and quantity of isolated DNA and consequently the low confidence in this negative assay result). Both β-HPV assays were positive for 19 specimens and negative for 90 specimens, giving 91.6 % observed agreement. The E1-based assay was able to detect additional 10 positive cases that had been reported by the RHA Kit as negative. Cohen’s kappa was determined to be 0.742 (CI_95_ = 0.594–0.890) indicating good agreement between these two assays. For further biomedical interpretation we considered cases detected by E1-based PCR assay as β-HPV-positive.

Dual infections with both α-HPV and β-HPV genera were not observed among 120 specimens evaluable for both α-HPV and β-HPV statuses. All 12 specimens, which were found positive for α-HPV genotypes, were β-HPV-negative, and conversely, all 30 specimens, which were found positive for β-HPV genotypes, were α-HPV-negative. Assuming that the distribution of detected α-HPV and β-HPV infections across these 120 specimens is independent, the probability of occurrence of dual α-HPV/β-HPV infections could be estimated as 100 × (12/120) × (30/120) = 2.5 %, and the binomial probability of finding exactly 0 dual α-HPV/β-HPV infections among 120 cases would be p(0) = 0.04792. Considering the relatively low p(0) value, our data do not support the assumption of independent occurrence of α-HPV and β-HPV infections and suggest that the dual infections by α-HPV and β-HPV genera occurs less frequently than expected by chance, if they occur at all in Bowen disease.

**Fig. 5:**
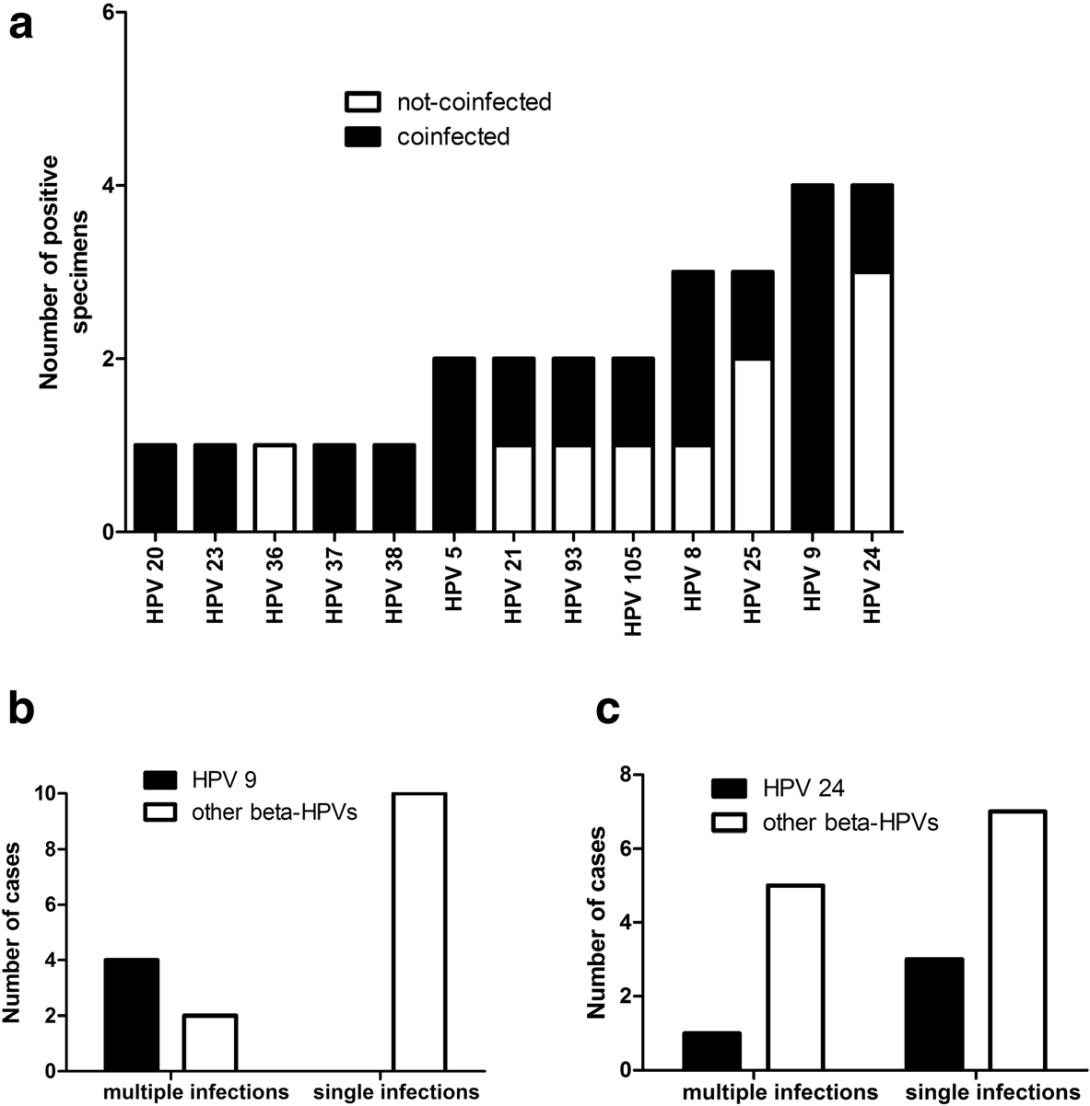
**a** Distribution of all β-HPV genotypes across 16 β-HPV-positive specimens for which specific β-HPV genotypes could be identified. Full bars: specimens with 2 or more β-HPV genotypes; empty bars: specimens with single β-HPV genotype. **b** Status of β-HPV infection (mixed vs single infection) across specimens with or without detected HPV 9. **c** Status of β-HPV infection (mixed vs single infection) across specimens with or without detected HPV 24

Next, the presence of mixed infections by two or more genotypes was examined in BD specimens infected exclusively by α-HPV or β-HPV genera. All of the 13 lesions positive for α-HPV were infected by single virus genotypes with following distribution: HPV 16: six lesions; HPV 33: two lesions; HPV 18, 56, 58, 66 and 81: one lesion each. In contrast, 16 β-HPV-positive specimens, for which virus genotype could be determined, displayed the following distribution of the number of distinct β-HPV genotypes: 10 specimens: 1 genotype; 2 specimens: 2 genotypes; 3 specimens: 3 genotypes, and 1 specimen: 4 distinct β-HPV genotypes. The proportion of β-HPV-positive specimens infected with multiple β-HPV types was 37.5 % (CI_95_ = 18.5–61.4 %) and the average number of β-HPV genotypes per specimen was found to be 1.69. Interestingly, all specimens positive for multiple β-HPV genotypes corresponded to morphologically single BD lesions. The distribution of β-HPV genotypes detected in these 16 specimens is presented in Fig. 5a.

In order to assess whether mixed infections by several β-HPV genotypes occur more often than expected by chance, we performed permutation test, in which 10.000 permutation replicates were constructed for 16 specimens with the same distribution of β-HPV genotypes as depicted in Fig. 5a. The proportion of specimens with two or more distinct β-HPV genotypes in permutation replicates was found to be 0.485 (77604/160000), which would correspond to the hypothetical proportion of BD lesions with mixed β-HPV infections, if the infections by β-HPV were independent events. The observed proportion of mixed β-HPV infections 0.375 is not significantly different from the hypothetical proportion 0.485 (z-test for one proportion, *p* = 0.3786); therefore, we cannot reject the hypothesis that β-HPV infections are independent events which co-occur as expected by chance.

Intriguingly, however, HPV 9 has been detected in 4 specimens that were all infected by at least one another β-HPV genotype (Fig. 5b), while HPV 24 has been detected in 3 BD specimens as the only infecting β-HPV genotype and in 1 BD specimen co-occurring with another genotype (Fig. 5c). The association between the status of infection (mixed infections vs single infection) and the presence or absence of HPV 9 is statistically significant (two-tailed *p* = 0.0082, Fisher’s exact test), which supports the hypothesis that HPV 9 co-occurs in BD lesions with other β-HPV genotypes more often than expected by chance. Consequently, HPV 9-positive BD lesions are more likely to display mixed infections by several β-HPV genotypes than BD lesions positive for any β-HPV genotype other than HPV 9 (odds ratio 37.8; CI_95_ = 1.493–957.2). In contrast with HPV 9, the association between the HPV 24 and the status of infection (mixed infections vs single infection) is not significant (two-tailed *p* = 1.0000, Fisher’s exact test).

P16 expression was found positive in 22/30 (73.3 %, CI95 = 55.6–85.8 %) of β-HPV positive BD lesions, including all 4 specimens positive for HPV 5 or HPV 8, which have been previously reported by Akgül et al. as the “high-risk β-HPV genotypes” [38]. However, p16 expression was also found positive in 67/78 (85.9 %, CI_95_ = 76.5–91.9) of β-HPV negative BD lesions.

The overall prevalence of HPV-positivity (α and β genera combined) in BD cases in the studied population can be estimated as 34.7 % (CI_95_ = 26.8–43.5 %), which is significantly less than 50 % (one-tailed *p* = 0.0005). Consequently, the majority of BD cases in the studied population are not infected with high risk α-HPV or β-HPV.

The results presented in Table 1 suggest: (i) a higher prevalence of HPV-positive cases in sun-protected sites (11/26; 42.3 %) relative to sun-exposed sites (28/87; 32.2 %); (ii) a lower prevalence of solar elastosis in HPV-positive (21/42; 50 %) relative to HPV-negative (48/79; 60.8 %) specimens; (iii) a higher proportion of specimens with koilocyte-like changes in HPV-positive (21/42; 50 %) compared to HPV-negative (29/79; 36.7 %) biopsies; and (iv) a higher proportion of papillomatosis-positive cases in HPV-positive (17/41; 41.4 %) over HPV-negative (25/79; 31.6 %) cases. While none of these differences was found to be statistically significant, some of them are consistent with a biological background and consequently plausible (e.g. higher prevalence of koilocyte-like changes or papillomatosis in HPV-positive specimens).

The results demonstrated that there is no appreciable association between the p16-expression and the HPV DNA status (phi = - 0.08). The proportions of p16-positive cases in HPV-negative and HPV-positive BD specimens were similar (87.1 % vs 80.0 %; *p* =0.4247). The datasets supporting the conclusions of this article are included in Additional file 2.

## Discussion

The causative role of β-papillomavirus infection in the development of extragenital cSCC has been suggested decades ago [39]; however, it has not yet been widely accepted. Nevertheless, the growing body of evidence supports the involvement of β-HPV in the development of cSCC at least in epidermodysplasia verruciformis patients and at least indirectly, via the inhibition of UV-induced apoptosis and/or impaired DNA repair, or other mechanisms [40]. Consistent with these findings, the International Agency for Research on Cancer (IARC) classified two β-HPV genotypes HPV 5 and HPV 8 as possibly carcinogenic to humans (all other β-HPV and γ-HPV genotypes are presently listed as agents “not classifiable as to their carcinogenicity to humans”) [41].

Results published by other investigators reported a highly variable prevalence of HPV-positivity in malignant and pre-malignant cutaneous lesions, depending on the ethnicity and immune status of the study population, and methods used for the HPV detection [3–17]. Likewise, the spectrum of detected HPV genotypes varies considerably across different studies, which is consistent with the notion that β-HPV genotypes differ in their carcinogenicity [40, 41]. Our study is, to the best of our knowledge, the first study with more extensive sample size that reports (i) the prevalence of α- and β-papillomavirus genotypes, (ii) the association of HPV status with p16 expression, and (iii) the clinicopathological findings in β-HPV-positive SCC in situ in Czech (Eastern European Caucasian) immunocompetent patients.

The overall prevalence of HR α-HPV and β-HPV positivity in BD cases, estimated by our study as 34.7 % (CI_95_ = 26.8–43.5 %), is consistent with several previous publications that reported 33–40 % of HPV-positive BD cases [12, 42, 43]. Likewise, the HPV prevalence of 34.7 % in determined in this study for immunocompetent BD patients is not substantially different from 40 % of HPV-positivity determined for immunocompromised BD patients in our previous report (Švajdler et al. Am J Dermatopathol, 2016, in press). In contrast, other investigators reported higher prevalence of HPV-positive BD cases (60–91 %) [5, 7, 8, 16], but all these studies examined only limited numbers of BD cases (10 to 62). The difference in HPV prevalence reported by us and other investigators may also reflect different HPV assay sensitivities and different patient populations. In order to account for different assay sensitivities we have employed two different PCR-based assays and found good inter-assay agreement (91.6 %) with Cohen’s kappa of 0.742. In addition, for further statistical processing we used the results from E1-based assay, which detected more positive cases than RHA Kit Skin (beta) assay and yet we report lower overall prevalence of HPV in our set of BD lesions. Consequently, we consider our results to be reliably reflecting HPV status of BD lesions in our target Eastern European population. In this study we found similar HPV prevalence in male and female patients (31.3 % vs 35.0 %), which differs from our previous report that identified higher HPV prevalence in female relative to male patients; however, this difference may be related to the different immune statuses of patient populations under examination.

The median age at BD diagnosis in our set of immunocompetent patients is 74 years, which is consistent with 73 years reported by a large population-based study of non-genital BD cases in a European population [44], but considerably higher than 63.5 years found in our recent study of immunocompromised East European BD patients (Švajdler,et al. Am J Dermatopathol, 2016, in press). Consequently, our results support earlier onset of BD in immunocompromised patients relative to immunocompetent patients from the same geographical population.

In our set of HPV-positive BD specimens, the β-papillomavirus genotypes predominated over α-papillomavirus genotypes, which is consistent with previous reports on BD/carcinoma in situ in both immunocompetent and immunocompromised patients [5–8, 16, 42, 43]. Nevertheless, α-HPV genotypes were detected in significant proportion of BD lesions (~10 % of all BD lesions and ~29 % of HPV-positive BD lesions), which is in line with seven previous studies that examined the presence of only α-HPV genotypes in BD and reported in average 14.7 % of the HPV-positive BD cases (range 4.8–39 %) [13, 15, 23, 24, 45–47].

In analogy with other viruses, mixed viral infections may be expected to result from co-infection or superinfection of susceptible cells with two or more HPV genotypes [48] and indeed, mixed infections by several α-HPV genotypes [49, 50] or β-HPV genotypes [10] are commonly found in various clinical settings. Consistent with these findings, 37.5 % (CI_95_ = 18.5–61.4 %) of our β-HPV positive specimens displayed mixed infections with 2 or more β-HPV genotypes; however, all 13 BD lesions positive for α-HPV were infected only by single α-HPV genotypes. This finding is somewhat conflicting with previous reports according to which mixed α-HPV cervical infections were observed in 20–50 % of HPV-positive women [49], because with such an occurrence of mixed infections, the probability of finding exactly 13 single α-HPV infections among 13 α-HPV positive biopsies would be rather low (0.0122–5.50 %). As a result, the prevalence of mixed α-HPV infections in α-HPV-positive BD lesions is likely lower than the reported prevalence of mixed α-HPV infections in cervical infections/cervical lesions. This finding may be biologically relevant, since mixed α-HPV infections of uterine cervix reportedly increase HPV persistence and oncogenic potential [51, 52], and this “potentiating effect” appears to be rarely if ever involved in the cutaneous SCC carcinogenesis.

Similarly, we have not found among our BD specimens any lesion with dual infection by α-HPV and β-HPV genera. This finding supports the conclusion that mixed α-HPV/β-HPV infections in extragenital/extraungual Bowen’s disease occur less frequently than would be expected by chance, if the infections by α-HPV and β-HPV were independent events not affecting each other. While mixed α-HPV/β-HPV infections have been previously reported Bowen’s disease, these findings were limited to genital [53] and ungual [54] cases which may reflect different environment and susceptibility of target cells to mixed genera infections. Consequently, mixed α-HPV/β-HPV infections are unlikely involved in the etiology of extragenital/extraungual Bowen’s disease in the studied East European population.

In contrast, we observed mixed infections by several β-HPV genotypes as relatively frequent finding in BD lesions (6/16 β-HPV-positive lesions displayed mixed infections with two or more β-HPV genotypes). This finding is etiologically interesting, since cSCCs are reportedly more likely to harbor 3 or more β-HPV genotypes within the same specimen than sun-protected control specimens [10]. While statistical analysis could not reject the hypothesis that β-HPV infections in BD specimens are independent events that co-occur as expected by chance, our analysis identified HPV 9 as the β-HPV genotype that tends to occur more often in BD with mixed β-HPV infections than expected by chance. We hypothesize that the infection of cells by HPV 9 facilitates subsequent infections or co-infections by other β-HPV genotypes, which may be relevant with respect to malignant transformation of keratinocytes and warrants future investigation.

Immunohistochemical p16 detection is often used as a surrogate marker for the high-risk α-HPV infections in anogenital or oropharyngeal premalignant and malignant lesions [21, 22]. In Bowen’s disease, the overexpression of p16 was reported by several studies, with proportions ranging from 58 to 100 % of cases [13, 23–30]. Consequently, the IHC staining for p16 has been suggested as a useful adjunctive test to support the diagnosis of BD in diagnostically challenging cases and to distinguish them from actinic keratosis or seborrhoeic keratosis [30]. However, these studies employed highly variable cut-off definitions of p16-positivity. For instance, when “diffuse” nuclear and cytoplasmic staining for p16 is classified as positive, 70–100 % of BD cases were classified as p16-positive [13, 23, 25, 30], which is consistent with our finding of 134/168 (79.86 %) p16-positive BD cases.

So far, only few studies have correlated p16 expression in BD with the HPV status. In the study reported by Willman et al., 28/32 BD cases (88 %) showed strong diffuse p16 staining (>90 % of cells), while HPV DNA was present in only 3/20 cases [23]. Reuschenbach et al. found diffuse p16-positivity in 38/41 lesions (92.7 %), but HPV was detected in only 16/41 lesions (39.0 %) [13]. Likewise, Murao et al. reported strong p16 staining (>50 % of cells) in 103 of 133 lesions (73.6 %) and HPV-positivity in only 11 (8.3 %) cases [24]. All 3 studies concluded that p16 overexpression in BD is unrelated to the HPV status, but they only investigated the presence of mucosal HPV types. In contrast, both mucosal and cutaneous HPV genotypes were considered in our series. We demonstrated that neither the presence of α-HPV types nor the presence of β-HPV types correlated with p16-expression. The exact mechanism responsible for p16 expression in BD is currently unknown. Interestingly, two studies reported that p16-expression correlated with the loss of pRb expression [23, 24]. An inverse pattern of p16 and pRb staining suggests that the p16 overexpression may be a response to the lack of functional pRb, which may be independent on the presence of high risk HPV.

**Fig. 6:**
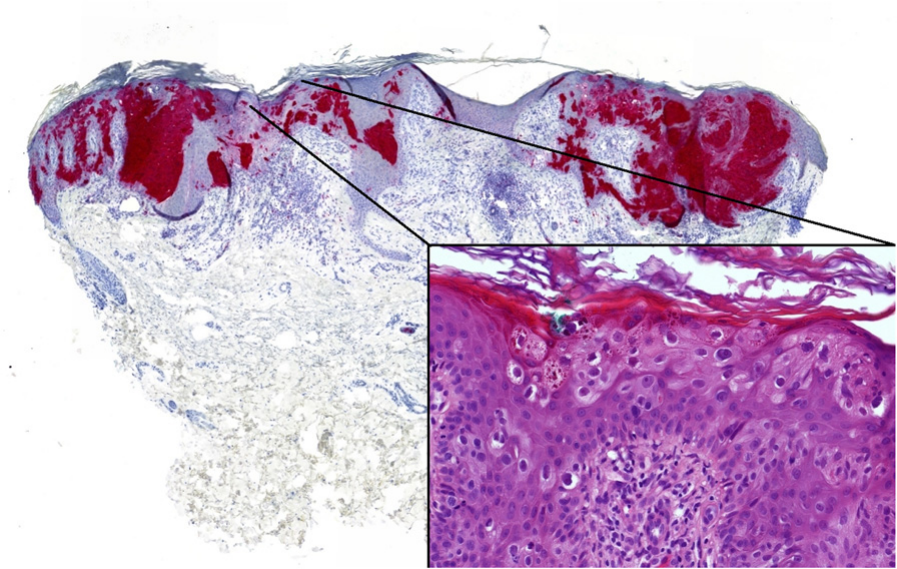
P16-positive “multicentric” Bowen’s disease showing well-defined “skip” areas and pagetoid spread (inset). Original magnification × 60 and × 400 (inset)

Recently, Gross et al. demonstrated that the vast majority of the “multicentric” SCCIS of the “Bowenoid” type (and its morphologic variants) showed diffuse p16 staining and suggested that HPV might be the factor responsible for many of the multicentric SCCIS in the same way than in multicentric anogenital SCCIS, in which “skip” areas between normal and abnormal epithelial cells has been strongly associated with HPV infections [55]. In our study, many BD lesions also showed “multicentric” features as defined by Gross et al. (including pagetoid spread and well-defined “skip” areas, Fig. 6). Twenty-one “multicentric” (multifocal) BD lesions from nine patients were examined and found to co-occur generally in the same anatomical sites, which might suggest common pathogenesis, such as viral infection. For ten p16-positive multifocal BD lesions the complete HPV status was available, and HPV could only be detected in five lesions. Based on this result, we believe that p16-positivity in a multifocal or multicentric BD lesion is not predictive for the presence of HPV.

Koilocyte-like changes and papillomatosis represent two features that are often present in HPV-associated lesions, such as verrucas or condylomas. While we detected these changes more frequently in HPV-positive than in HPV-negative BD specimens, the differences between proportions were not statistically significant; therefore we cannot support these features as reliable discriminants between HPV-positive and HPV-negative BD cases.

The mechanisms responsible for HPV driven malignant transformation of cutaneous keratinocytes are not yet fully understood and further investigation in that matter is warranted (reviewed by Quint et al [2]). Nevertheless, the epidemiological and experimental evidence suggests the role of combined effects of ultraviolet radiation, immunosuppression and β-papillomavirus infection. One of the possible interactions between HPV and UV can be explained by UV-induced T cell-mediated local immunosuppression [56] that may decrease the chances of virus clearance and increase the probability of multiple (mixed) HPV infections. This mechanism was supported by reports that found a higher prevalence of HPV infections in sun-exposed relative to sun-protected sites [9, 10]; however our results suggest a lower HPV prevalence in sun-exposed relative to sun-protected sites. Considering this finding we speculate that tumor development in sun-protected areas is more dependent on the co-carcinogenic effects of β-HPV than in sun-exposed areas, where the higher doses of UV radiation may be able to induce malignant transformation even without HPV-mediated co-carcinogenesis.

## Conclusions

In summary, approximately one third of BD lesions in our Czech (Caucasian Eastern European) population are HPV-associated. In the HPV-positive lesions β-HPV genotypes predominated, although high- and low-risk mucosal HPV types were detected in a significant proportion of cases. The presence of the p16-expression, koilocyte-like change or papillomatosis is not predictive for the presence of neither α-HPV types nor β-HPV types in extragenital/extraungual Bowen’s disease. Our study further supports the hypothesis that HPV infection might serve as an important co-factor in the carcinogenesis of BD and cSCC and identifies HPV 9 as a possibly significant β-HPV genotype with respect to mixed β-HPV infections and keratinocyte carcinogenesis.

## Abbreviations

HPV, human papilloma virus; BD, Bowen’s disease; cSCC, cutaneous squamous cell carcinoma; EV, epidermodysplasia verruciformis; HR, high risk; IHC, immunohistochemistry; PCR, polymerase chain reaction; pRb, retinoblastoma protein

## Additional files

Additional file 1: Summary of demography data and clinicopathological findings for 169 Bowden’s disease biopsy specimens. (DOCX 13 kb) Additional file 2: The datasets supporting the conclusions of the article. (XLSX 19 kb)
