# Supplementary material for: Human papillomavirus infection and p16 expression in the immunocompetent patients with extragenital/extraungual Bowen’s disease
Source: Diagn Pathol. 2016 Jun 24;11:53. doi: 10.1186/s13000-016-0505-3 (PMC4919835; doi:10.1186/s13000-016-0505-3)
Supplement: Additional file 1: — Summary of demography data and clinicopathological findings for 169 Bowden’s disease biopsy specimens. (DOCX 13 kb) [file 13000_2016_505_MOESM1_ESM.docx]

**Human papillomavirus infection and p16 expression in the immunocompetent patients with extragenital/extraungual Bowen's disease.**

# ****Additional File****

# ****Supplemental Table** Summary of demography data and clinicopathological findings for 169 Bowden's disease biopsy specimens**

|  | Female specimens | Male specimens | p-value  (Statistical test) |
| --- | --- | --- | --- |
| N | 75 | 94 | 0.1440  (one-sample z-test p-value for H_0_: equal proportion of male and female specimens) |
| **Mean Age ±SD**  **(first lesion dg)** | 73.8 **± 12.7** | 72.8 **± 9.1** | 0.5972  (Welch's corrected t-test) |
| Head&neck BD | 47 | 52 | 0.1797 |
| Upper extremity BD | 7 | 11 | (Chi-square test) |
| Trunk BD | 4 | 13 |  |
| Lower extremity BD  Unknown | 14  3 | 11  7 |  |
| P16-positive BD  P16-negative BD  P16-unknown | 62  13  0 | 72  21  1 | 0.4442  (Fisher's exact test) |
| HR α-HPV status  positive  negative  not evaluable | 7  50  18 | 5  59  30 | 0.5453  (Fisher's exact test) |
| β-HPV status  positive  negative  not evaluable | 14  42  19 | 16  48  30 | 1.00  (Fisher's exact test) |
| Koilocyte-like changes  positive  negative | 35  40 | 36  58 | 0.3467  (Fisher's exact test) |
| Solar elastosis  positive  negative | 43  32 | 50  44 | 0.6420  (Fisher's exact test) |
| Papillomatosis  positive  negative  unknown | 24  51  0 | 34  59  1 | 0.6249  (Fisher's exact test) |
